# Admission platelet-to-hemoglobin ratio and 1-year outcomes after percutaneous coronary intervention: a multicenter registry cohort study

**DOI:** 10.3389/fcvm.2026.1981126

**Published:** 2026-10-09

**Authors:** Jeong Tae Byoun, Donghyeon Joo, Sungho Jo, Jae Young Cho, Kyeong Ho Yun

**Affiliations:** Department of Cardiovascular Medicine, Regional Cardiocerebrovascular Center, Wonkwang University Hospital, Iksan, Republic of Korea

**Keywords:** anemia, major adverse cardiovascular events, percutaneous coronary intervention, platelet, platelet-to-hemoglobin ratio, risk stratification

## Abstract

**Background:**

The platelet-to-hemoglobin ratio (PHR) is a proposed prognostic marker in cardiovascular disease, but it has not been evaluated against a broad major adverse cardiovascular events (MACE) endpoint in a dedicated percutaneous coronary intervention (PCI) cohort, and whether it adequately summarizes its two components remains untested.

**Methods:**

We analyzed 1,551 patients pooled from two prospective Korean drug-eluting stent registries (2016–2022). The PHR was calculated as platelet count (×10⁹/L) divided by hemoglobin (g/L). The primary endpoint was 1-year MACE, with follow-up censored at 365 days, a composite of all-cause death, myocardial infarction, stroke, repeat revascularization, or heart failure hospitalization. Multivariable Cox models included eight prespecified clinical covariates; the functional form was examined with restricted cubic splines, and the PHR was decomposed using nested models and a log-scale ratio-constraint test.

**Results:**

MACE occurred in 107 patients (6.9%) within 1 year. A higher PHR was independently associated with MACE (adjusted hazard ratio [HR] per 1-standard deviation, 1.21; 95% confidence interval [CI], 1.05–1.40; *P* = 0.008), with no evidence of nonlinearity (*P* = 0.700). Adding the PHR improved the clinical model (likelihood-ratio *P* = 0.013); the ratio constraint was not rejected (*P* = 0.765), and a single log-PHR term retained 98.7% of the two-component likelihood-ratio *χ*^2^ increment over the clinical model in this cohort (bootstrap 95% CI, 21.8%–100%). The PHR was associated with all-cause and cardiovascular death (both HR, 1.28), paralleling hemoglobin. Repeat revascularization was associated with platelet count (HR, 1.39; *P* = 0.014), but not significantly with PHR (HR, 1.24; *P* = 0.081). Risk was concentrated in patients with both anemia and a platelet count at or above the median (adjusted HR, 1.82; 95% CI, 1.05–3.15). The gain in discrimination was negligible (*Δ*AUC, +0.003; 95% CI, −0.003 to +0.012), and net benefit was virtually unchanged.

**Conclusions:**

The admission PHR was independently associated with 1-year MACE after PCI, with no evidence of nonlinearity, and its log-scale component effects were compatible with the ratio constraint in this cohort. Given the negligible gain in discrimination and net benefit, the PHR is an independent prognostic marker rather than a tool for risk reclassification.

**Registry registration:** Clinical Research Information Service KCT0003392 and KCT0005008.

## 1 Introduction

Despite continued advances in revascularization techniques and adjunctive pharmacotherapy, a substantial proportion of patients undergoing percutaneous coronary intervention (PCI) experience adverse cardiovascular events within the first year after the index procedure (1). This persistent residual risk has sustained interest in simple, inexpensive, and universally available markers for early risk stratification. Indices derived from the routine complete blood count (CBC), such as the neutrophil-to-lymphocyte ratio and the platelet-to-lymphocyte ratio, have been extensively investigated in patients undergoing PCI (2, 3). Among the individual CBC components, a higher baseline platelet count has been associated with thrombotic complications and adverse outcomes after primary PCI (4), whereas a lower hemoglobin concentration is a consistent predictor of mortality after PCI, reflecting impaired oxygen delivery, comorbidity burden, and bleeding susceptibility (5).

The platelet-to-hemoglobin ratio (PHR) integrates these two opposing prognostic signals into a single index. In the first report from the Clinical Outcomes and Risk Factors of Patients with Coronary Heart Disease after PCI (CORFCHD-PCI) study of 6,046 patients, Zheng et al. found that the PHR predicted long-term adverse outcomes after PCI with better discrimination than either platelet count or hemoglobin alone (6). Subsequent studies have associated an elevated PHR with long-term all-cause mortality in patients with coronary artery disease and congestive heart failure (7), in-hospital mortality in ST-segment elevation myocardial infarction (STEMI) (8), long-term mortality in acute coronary syndrome, particularly among patients with diabetes mellitus (9), and in-hospital 3-point major adverse cardiovascular events (MACE) after coronary artery bypass grafting (CABG) (10).

Nevertheless, several important gaps remain. First, beyond the initial brief report (6), the PHR has not been evaluated against a broad MACE endpoint in a dedicated PCI cohort with comprehensive multivariable adjustment, and external validation across diverse populations and practice settings remains limited. Second, and more fundamentally, a ratio index implicitly assumes that its numerator and denominator carry prognostic effects of equal magnitude and opposite direction on the logarithmic scale; to our knowledge, no previous study has formally tested whether the PHR adds prognostic information beyond its components or whether a single ratio term adequately captures their joint information—an assumption that, when violated, can distort the apparent association of ratio variables (11). Third, previously proposed cutoff values vary widely (1.69 to 2.2) (7–9), the reported dose–response relationship has been inconsistent across endpoints (7, 9), and both components—and hence the PHR distribution itself—differ substantially by sex, raising questions about the transportability of any single threshold.

Accordingly, we performed a pooled analysis of two prospective Korean drug-eluting stent registries to examine the association between the admission PHR, analyzed as a continuous variable, and 1-year MACE after PCI. We further conducted a formal decomposition to determine whether the PHR conveys prognostic information beyond hemoglobin and platelet count individually and whether it adequately summarizes their joint information, and we explored endpoint-specific and subgroup patterns of this association.

## 2 Materials and methods

### 2.1 Study population

This study was a pooled, patient-level analysis of two prospective drug-eluting stent registries conducted at three tertiary cardiovascular centers in Jeonbuk Province, Republic of Korea. The Jeonbuk SYNERGY stent registry (KCT0003392) enrolled all-comer patients with coronary artery disease who underwent successful implantation of the thin-strut everolimus-eluting SYNERGY stent between 2016 and 2019. The Jeonbuk Orsiro stent registry (KCT0005008) enrolled all-comer patients who underwent successful percutaneous coronary intervention (PCI) with the ultrathin-strut sirolimus-eluting Orsiro stent between 2019 and 2022; in this registry, enrollment of patients with diabetes mellitus or prediabetes was encouraged, but enrollment was not restricted according to glycemic status.

A total of 1,586 patients were enrolled in the two registries. No additional clinical exclusion criteria were applied for the present analysis beyond the availability of the required variables. Thirty-five patients (2.2%) were excluded solely because of missing model covariates: 1 patient with missing clinical variables and 34 patients with missing echocardiographic or laboratory variables. The final study population therefore comprised 1,551 patients with available admission hemoglobin and platelet counts and all primary-model covariates (Figure 1). Clinical follow-up was scheduled at 12 ± 2 months after the index procedure. The primary time horizon was 1 year, defined as 365 days after the index procedure; follow-up was administratively censored at day 365, and follow-up duration was defined as the time from the index procedure to the first MACE, the last clinical contact, or day 365, whichever came first. The study protocol was approved by the Institutional Review Board of Wonkwang University Hospital (WKUH 201604-HR-036 and WKUH 2019-01-027-002), and all participants provided written informed consent at the time of enrollment in the respective registries. The study was conducted in accordance with the principles of the Declaration of Helsinki.

**Figure 1:**
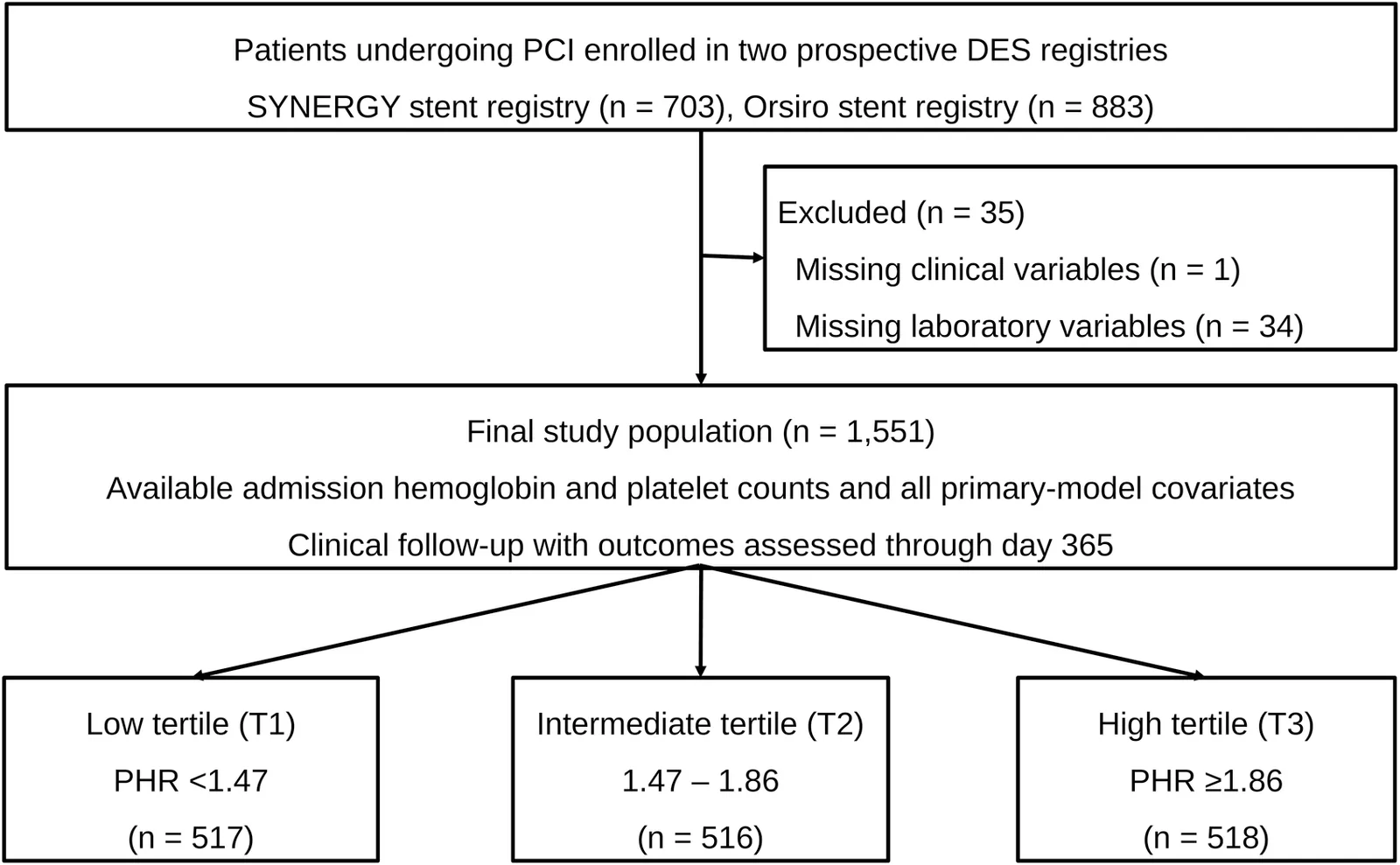
Study flow diagram. DES, drug-eluting stent; MACE, major adverse cardiovascular events; PCI, percutaneous coronary intervention; PHR, platelet-to-hemoglobin ratio.

### 2.2 Endpoints and definitions

The PHR was calculated as the platelet count (×10⁹/L) divided by the hemoglobin concentration (g/L) (6). All laboratory parameters, including the complete blood count used to derive the PHR, were obtained from admission blood samples drawn before the index procedure.

The primary endpoint was MACE, defined as a composite of all-cause death, myocardial infarction (MI), stroke, target vessel revascularization (TVR), non-TVR, or hospitalization for heart failure, analyzed as the time from the index procedure to the first occurrence of any component. Prespecified endpoint-specific analyses examined all-cause death, cardiovascular death, a composite of cardiovascular death, MI, or stroke, the individual components of MACE, and any repeat revascularization (TVR or non-TVR); for non-fatal endpoints, cause-specific analyses were performed with censoring at the time of death. Death, MI, stroke, and hospitalization for heart failure were defined in accordance with the 2017 Cardiovascular and Stroke Endpoint Definitions for Clinical Trials (12), and revascularization endpoints, including TVR and non-TVR, followed the Academic Research Consortium-2 consensus definitions (13).

Anemia was defined according to the World Health Organization criteria as a hemoglobin concentration <13 g/dL in men and <12 g/dL in women (14). The estimated glomerular filtration rate (eGFR) was calculated using the 2021 Chronic Kidney Disease Epidemiology Collaboration creatinine equation, and chronic kidney disease was defined as an eGFR <60 mL/min/1.73 m^2^ (15). Left main or multivessel disease was defined as significant stenosis of the left main coronary artery or of two or more major epicardial coronary arteries. A potent P2Y12 inhibitor was defined as ticagrelor or prasugrel prescribed at discharge. The recorded antiplatelet regimen at discharge was classified as dual antiplatelet therapy (aspirin plus a P2Y12 inhibitor), a P2Y12 inhibitor without aspirin, aspirin without a P2Y12 inhibitor, or neither recorded antiplatelet agent. Because oral anticoagulant use was not captured, these categories describe the recorded antiplatelet regimen rather than the complete antithrombotic regimen.

### 2.3 Statistical analysis

Continuous variables are presented as mean ± standard deviation or median [interquartile range], and categorical variables as number (percentage). Baseline characteristics were compared across PHR tertiles using one-way analysis of variance, the Kruskal–Wallis test, or the chi-square test, as appropriate. Tertiles were used for descriptive purposes; the prespecified primary analysis modeled the PHR as a continuous variable. Cumulative incidence was estimated by the Kaplan–Meier method and compared using the log-rank test.

The association between the PHR and MACE was evaluated using Cox proportional hazards regression with Efron's method for ties. The multivariable model included age, sex, hypertension, diabetes mellitus, MI presentation, left main or multivessel disease, eGFR, and left ventricular ejection fraction, and hazard ratios (HRs) for the PHR are reported per 1-standard deviation (0.58) increase. Covariates were prespecified on clinical grounds as established prognostic factors for MACE after PCI and factors plausibly associated with the PHR, while limiting model complexity to approximately 10 or more outcome events per parameter (107 events and 9 parameters in the primary model). The functional form of the PHR was examined using restricted cubic splines with four knots placed at the 5th, 35th, 65th, and 95th percentiles, and departure from linearity was tested with the likelihood-ratio test comparing the spline model with the linear model. HRs across PHR tertiles and the test for trend, obtained by entering the tertile as an ordinal term, are additionally reported for descriptive purposes. The proportional hazards assumption was assessed for the PHR and for each adjustment covariate using scaled Schoenfeld residuals (rank-transformed time); where non-proportionality was detected for an adjustment covariate, a sensitivity analysis with baseline hazards stratified by categories of that covariate was performed. Because the proportional hazards assumption pertains to the Cox model, the robustness of the primary association was additionally examined with a piecewise Cox model allowing separate PHR coefficients before and after 180 days (a time-varying coefficient), and with differences in restricted mean event-free time at 365 days, which do not require proportional hazards. Additional sensitivity analyses of the primary association included stratification of the baseline hazard by registry or by center, or inclusion of registry as a covariate; further adjustment for smoking, previous PCI, and body mass index, for aspirin use at discharge, and for log-transformed white blood cell count and, in the subset with available values, log-transformed high-sensitivity C-reactive protein (hs-CRP); and analysis of a sex-standardized PHR (z-score within each sex). Registry membership reflected differences in calendar period, stent platform, and enrollment strategy; its influence was examined using registry-adjusted and registry-stratified sensitivity analyses. Center heterogeneity was assessed with a center-stratified Cox model allowing a separate baseline hazard for each center. Cluster-robust variance estimation was not used because there were only three centers; stratification addresses baseline-hazard heterogeneity but does not directly estimate within-center correlation.

To compare the prognostic contributions of the PHR and its components, we evaluated the clinical model alone and models additionally containing hemoglobin, platelet count, both components, or the PHR. Likelihood-ratio tests compared each extended model with the clinical model; additional nested comparisons evaluated each component after inclusion of the other. Model fit and discrimination were summarized by the likelihood-ratio chi-square, the Akaike information criterion, and Harrell's C-index. Because the logarithm of the PHR equals the difference between log-platelet count and log-hemoglobin, the implicit ratio constraint (equal and opposite coefficients of the log-transformed components) was formally tested with a 1-degree-of-freedom likelihood-ratio test comparing the model containing a single log-PHR term with the model containing both log-transformed components, and the adequacy of the ratio was quantified as the proportion of the two-component likelihood-ratio chi-square increment over the clinical model retained by the single log-PHR term. Uncertainty around the deviation from the ratio constraint (the sum of the log-platelet count and log-hemoglobin coefficients) and around the adequacy index was quantified with Wald and percentile bootstrap 95% confidence intervals (1,000 resamples). Discrimination for 1-year MACE was further assessed using Uno's inverse-probability-of-censoring–weighted time-dependent area under the receiver operating characteristic curve (AUC) at 365 days; the difference in AUC between models was evaluated with percentile bootstrap confidence intervals, and optimism-corrected AUCs were obtained by bootstrap resampling (600 replicates). Calibration at 365 days was assessed by comparing predicted and observed (Kaplan–Meier) risks across deciles of predicted risk and by the optimism-corrected calibration slope (300 bootstrap resamples), and clinical usefulness was evaluated by decision-curve analysis, in which the net benefit of classifying patients as high risk across a range of threshold probabilities was estimated with Kaplan–Meier–based event probabilities to account for censoring.

Endpoint-specific associations were examined with cause-specific Cox models using the same covariates, censoring non-fatal endpoints at the time of death. The joint association of the PHR components was illustrated by classifying patients into four groups according to the presence of anemia and a platelet count below versus at or above the median, with adjusted HRs estimated against the group without either feature and multiplicative interaction tested on both the binary and continuous scales. Prespecified subgroup analyses were performed with the stratifying variable omitted from the adjustment set within its own strata, and effect modification was assessed using interaction terms. Analyses were based on complete cases (2.2% excluded); secondary and subgroup analyses were considered exploratory and were not adjusted for multiplicity. A two-sided P value <0.05 was considered statistically significant. All analyses were performed using SPSS version 30.0 (IBM Corp., Armonk, NY, USA) and Python 3.13.5 (Python Software Foundation, Wilmington, DE, USA).

## 3 Results

### 3.1 Baseline characteristics

The mean PHR was 1.74 ± 0.58 [median, 1.65 (interquartile range, 1.37–1.99)], and patients were classified into tertiles (<1.47, 1.47–1.86, and ≥1.86) for descriptive purposes. Baseline characteristics according to PHR tertiles are shown in Table 1. The proportion of men decreased markedly across tertiles (89.9%, 79.3%, and 60.0%; *P* < 0.001). Patients in the highest tertile were older, had a lower body mass index and eGFR, and showed higher white blood cell counts and hs-CRP concentrations (all *P* ≤ 0.027), whereas STEMI was less frequent (28.2%, 23.3%, and 21.6%; P for diagnosis = 0.012). Angiographic and procedural characteristics and discharge medications were generally balanced across tertiles. Dual antiplatelet therapy was prescribed at discharge in 87.4% of patients and a P2Y12 inhibitor without aspirin in 12.0%, with no difference across tertiles (*P* = 0.47); a P2Y12 inhibitor without aspirin was almost exclusively used in the Orsiro registry (21.7% vs 0.1% in the SYNERGY registry), indicating a difference in recorded antiplatelet practice between registry periods. Compared with the 1,551 included patients, the 35 patients excluded for missing covariates were more often women, had diabetes, and were enrolled in the Orsiro registry, and less often presented with acute MI, whereas no statistically significant differences were observed in hemoglobin, platelet count, the PHR, or the incidence of MACE; the small excluded group limits the precision of these comparisons (Supplementary Table S1).

**Table 1:** Baseline characteristics according to tertiles of the platelet-to-hemoglobin ratio.

Variable	Tertile 1 (<1.47) (*n* = 517)	Tertile 2 (1.47–1.86) (*n* = 516)	Tertile 3 (≥1.86) (*n* = 518)	P value
Age, years	64.1 ± 10.6	64.2 ± 10.5	65.7 ± 11.2	0.027
Male sex	465 (89.9)	409 (79.3)	311 (60.0)	<0.001
Body mass index, kg/m^2^	25.2 ± 3.1	25.0 ± 3.1	24.5 ± 3.7	0.003
Hypertension	322 (62.3)	320 (62.0)	351 (67.8)	0.094
Diabetes mellitus	256 (49.5)	242 (46.9)	250 (48.3)	0.702
Current smoker	163 (31.7)	157 (30.4)	148 (28.6)	0.568
Previous PCI	61 (11.8)	46 (8.9)	41 (7.9)	0.087
Diagnosis				0.012
Stable angina	96 (18.6)	103 (20.0)	76 (14.7)	
Unstable angina	166 (32.1)	186 (36.0)	194 (37.5)	
NSTEMI	109 (21.1)	107 (20.7)	136 (26.3)	
STEMI	146 (28.2)	120 (23.3)	112 (21.6)	
Hemoglobin, g/dL	14.7 ± 1.7	14.0 ± 1.5	12.9 ± 2.0	<0.001
Platelet count, ×10^3^/μL	180.3 ± 31.7	231.7 ± 27.5	296.9 ± 57.6	<0.001
White blood cell count, ×10^3^/μL	8.3 ± 3.1	8.4 ± 3.0	9.3 ± 3.6	<0.001
hs-CRP, mg/L	1.32 [0.55–3.25]	1.27 [0.67–3.41]	1.75 [0.80–5.05]	<0.001
Glucose, mg/dL	158.0 ± 69.7	149.8 ± 65.3	152.1 ± 73.4	0.158
Total cholesterol, mg/dL	175.5 ± 46.5	179.7 ± 46.2	180.7 ± 48.5	0.199
Triglyceride, mg/dL	138.00 [92.50–194.50]	129.50 [92.00–197.00]	126.00 [89.00–188.00]	0.356
LDL cholesterol, mg/dL	106.7 ± 41.6	109.9 ± 39.0	109.6 ± 41.7	0.424
HDL cholesterol, mg/dL	43.5 ± 11.2	45.6 ± 12.4	44.6 ± 12.4	0.026
Creatinine, mg/dL	0.90 [0.79–1.08]	0.90 [0.75–1.04]	0.87 [0.70–1.09]	0.033
eGFR, mL/min/1.73 m^2^	86.0 ± 20.8	86.1 ± 20.8	81.8 ± 25.3	0.002
Ejection fraction, %	55.1 ± 10.9	56.4 ± 11.7	55.3 ± 11.2	0.158
LM or multivessel disease	238 (46.0)	217 (42.1)	243 (46.9)	0.247
Type B2/C lesion	385 (78.9)	391 (80.5)	385 (78.7)	0.765
Calcified lesion	40 (8.2)	45 (9.3)	35 (7.2)	0.489
Bifurcation lesion	76 (15.6)	73 (15.0)	75 (15.3)	0.971
Chronic total occlusion	27 (5.2)	24 (4.7)	28 (5.4)	0.848
In-stent restenosis	12 (2.5)	11 (2.3)	8 (1.6)	0.647
Intravascular ultrasound use	58 (11.9)	47 (9.7)	47 (9.6)	0.415
Number of stents	1.6 ± 0.9	1.5 ± 0.9	1.5 ± 0.8	0.303
Total stent length, mm	42.4 ± 28.7	42.0 ± 27.3	39.3 ± 25.7	0.136
Minimal stent diameter, mm	3.03 ± 0.43	3.01 ± 0.44	2.99 ± 0.42	0.307
Stent type				0.038
SYNERGY	211 (40.8)	251 (48.6)	237 (45.8)	
Orsiro	306 (59.2)	265 (51.4)	281 (54.2)	
Discharge medication				
Aspirin	447 (86.5)	447 (86.6)	464 (89.6)	0.233
P2Y12 inhibitor	514 (99.4)	514 (99.6)	513 (99.0)	0.497
Beta blocker	318 (62.5)	295 (57.8)	311 (61.2)	0.294
ACEI or ARB	388 (76.2)	370 (72.5)	384 (75.6)	0.352
Statin	504 (97.5)	508 (98.4)	505 (97.5)	0.476
Recorded antiplatelet regimen			0.473	
DAPT	446 (86.3)	447 (86.6)	462 (89.2)	
P2Y12 inhibitor without aspirin	68 (13.2)	67 (13.0)	51 (9.8)	
Aspirin without a P2Y12 inhibitor	1 (0.2)	0 (0.0)	2 (0.4)	
Neither recorded antiplatelet agent	2 (0.4)	2 (0.4)	3 (0.6)	

Values are mean ± standard deviation, median [interquartile range], or n (%). Percentages were calculated using the number of patients with available data for each variable. P values by one-way ANOVA, Kruskal–Wallis test, or chi-square test, as appropriate; for diagnosis and stent type, a single chi-square test across the categories is shown.

ACEI, angiotensin-converting enzyme inhibitor; ARB, angiotensin receptor blocker; DAPT, dual antiplatelet therapy; eGFR, estimated glomerular filtration rate; hs-CRP, high-sensitivity C-reactive protein; LDL, low-density lipoprotein; HDL, high-density lipoprotein; LM, left main; NSTEMI, non–ST-segment elevation myocardial infarction; PCI, percutaneous coronary intervention; STEMI, ST-segment elevation myocardial infarction.

### 3.2 Association between the PHR and 1-year MACE

Within 1 year (365 days) after the index procedure, MACE occurred in 107 patients (6.9%; Kaplan–Meier cumulative incidence, 7.0%). Protocol-scheduled 1-year follow-up was completed in 1,543 patients (99.5%). The remaining 8 patients (0.5%) were lost to follow-up: their last clinical contact occurred before day 305, and none had experienced MACE before that contact. In addition, 179 patients completed the scheduled visit between days 305 and 364 and had no subsequent contact; none of them had experienced MACE by the time of that visit. These 187 event-free patients were censored at their last clinical contact, which in all cases preceded day 365. Consequently, the status at day 365 was known for 1,364 patients (87.9%): 107 had experienced MACE by day 365 and 1,257 were event-free and remained under observation at day 365.

Clinical outcomes according to PHR tertiles are presented in Table 2A; MACE occurred in 5.0%, 6.6%, and 9.1% of patients in tertiles 1 to 3, respectively (log-rank *P* = 0.035). In the prespecified primary analysis, a higher PHR as a continuous variable was associated with an increased risk of MACE (unadjusted HR per 1-standard deviation increase, 1.36; 95% confidence interval [CI], 1.19–1.56; *P* < 0.001), and this association remained significant after multivariable adjustment (adjusted HR, 1.21; 95% CI, 1.05–1.40; *P* = 0.008; Table 2B). On restricted cubic spline analysis, there was no evidence of departure from linearity (P for nonlinearity = 0.700), and the linear model yielded the lowest Akaike information criterion (Figure 2). In the descriptive tertile analysis, the adjusted HR for the highest versus lowest tertile was 1.61 (95% CI, 0.97–2.68; *P* = 0.064; P for trend = 0.068), consistent in direction with the continuous analysis but attenuated by the loss of information inherent to categorization. On scaled Schoenfeld residuals, the proportional hazards assumption was satisfied for the PHR (*P* = 0.707) and for all adjustment covariates except MI presentation (*P* = 0.029; ejection fraction, *P* = 0.149); models with baseline hazards stratified by MI presentation (HR, 1.21; 95% CI, 1.05–1.40) or by MI presentation and ejection fraction category (HR, 1.22; 95% CI, 1.06–1.41) yielded virtually identical estimates. A piecewise model allowing the PHR coefficient to differ before and after 180 days showed similar HRs in the two periods (1.20 [95% CI, 1.01–1.43] for days 0–180 and 1.24 [95% CI, 0.99–1.56] for days 181–365; P for difference = 0.82), and the restricted mean event-free time at 365 days, which does not rely on proportional hazards, was 352.2, 350.7, and 344.6 days in tertiles 1 to 3 (difference, tertile 3 vs 1, −7.6 days; 95% CI, −15.8 to +0.6; Supplementary Table S2). Effect estimates were directionally similar across sensitivity analyses (Supplementary Table S3), including stratification by registry (HR, 1.22; 95% CI, 1.06–1.41) or by center (HR, 1.21; 95% CI, 1.05–1.40), additional adjustment for smoking, previous PCI, and body mass index (HR, 1.23; 95% CI, 1.06–1.43) or for aspirin use at discharge (HR, 1.22; 95% CI, 1.06–1.41), and analysis of the sex-standardized PHR (HR per sex-specific SD, 1.20; 95% CI, 1.05–1.38). Additional adjustment for log white blood cell count modestly attenuated the association (HR, 1.19; 95% CI, 1.03–1.39), and in the subset with available hs-CRP (*n* = 1,303; 97 events), adjustment for log hs-CRP attenuated the HR from 1.20 (95% CI, 1.03–1.39) to 1.16 (95% CI, 1.00–1.35; *P* = 0.054), with the confidence interval reaching the null.

**Table 2:** Clinical outcomes at 1-year and association of the platelet-to-hemoglobin ratio.

A. Outcomes according to PHR tertiles				
Outcome	Tertile 1 (*n* = 517)	Tertile 2 (*n* = 516)	Tertile 3 (*n* = 518)	Log-rank P
MACE	26 (5.0)	34 (6.6)	47 (9.1)	0.035
KM estimate at 365 days, % (95% CI)	5.1 (3.5–7.3)	6.7 (4.8–9.2)	9.2 (7.0–12.0)	
All-cause death	8 (1.5)	11 (2.1)	16 (3.1)	0.238
Cardiovascular death	8 (1.5)	10 (1.9)	16 (3.1)	0.209
Myocardial infarction	1 (0.2)	7 (1.4)	4 (0.8)	0.103
Stroke	8 (1.5)	8 (1.6)	10 (1.9)	0.849
Target vessel revascularization	3 (0.6)	9 (1.7)	7 (1.4)	0.223
Non-target vessel revascularization	6 (1.2)	8 (1.6)	12 (2.3)	0.324
Any repeat revascularization	9 (1.7)	16 (3.1)	17 (3.3)	0.238
Heart failure hospitalization	2 (0.4)	3 (0.6)	7 (1.4)	0.168

B. Association between PHR and MACE				
PHR	Unadjusted HR (95% CI)	P	Adjusted HR (95% CI)	P
Per 1-SD increase (0.58)	1.36 (1.19–1.56)	<0.001	1.21 (1.05–1.40)	0.008
Per 1-unit increase	1.70 (1.35–2.15)	<0.001	1.40 (1.09–1.79)	0.008
Tertile 1	1 (reference)		1 (reference)	
Tertile 2	1.32 (0.79–2.19)	0.291	1.44 (0.86–2.40)	0.169
Tertile 3	1.84 (1.14–2.97)	0.013	1.61 (0.97–2.68)	0.064
P for trend		0.011		0.068

Values are number of events (%) unless stated. Event counts, log-rank tests, Cox analyses, and Kaplan–Meier (KM) estimates refer to 1 year (365 days) after the index procedure, with follow-up censored at day 365. Adjusted models included age, sex, hypertension, diabetes mellitus, myocardial infarction presentation, left main or multivessel disease, estimated glomerular filtration rate, and left ventricular ejection fraction. P for trend was obtained by entering tertile as an ordinal term.

CI, confidence interval; HR, hazard ratio; MACE, major adverse cardiovascular events (all-cause death, myocardial infarction, stroke, any revascularization, or heart failure hospitalization); SD, standard deviation.

**Figure 2:**
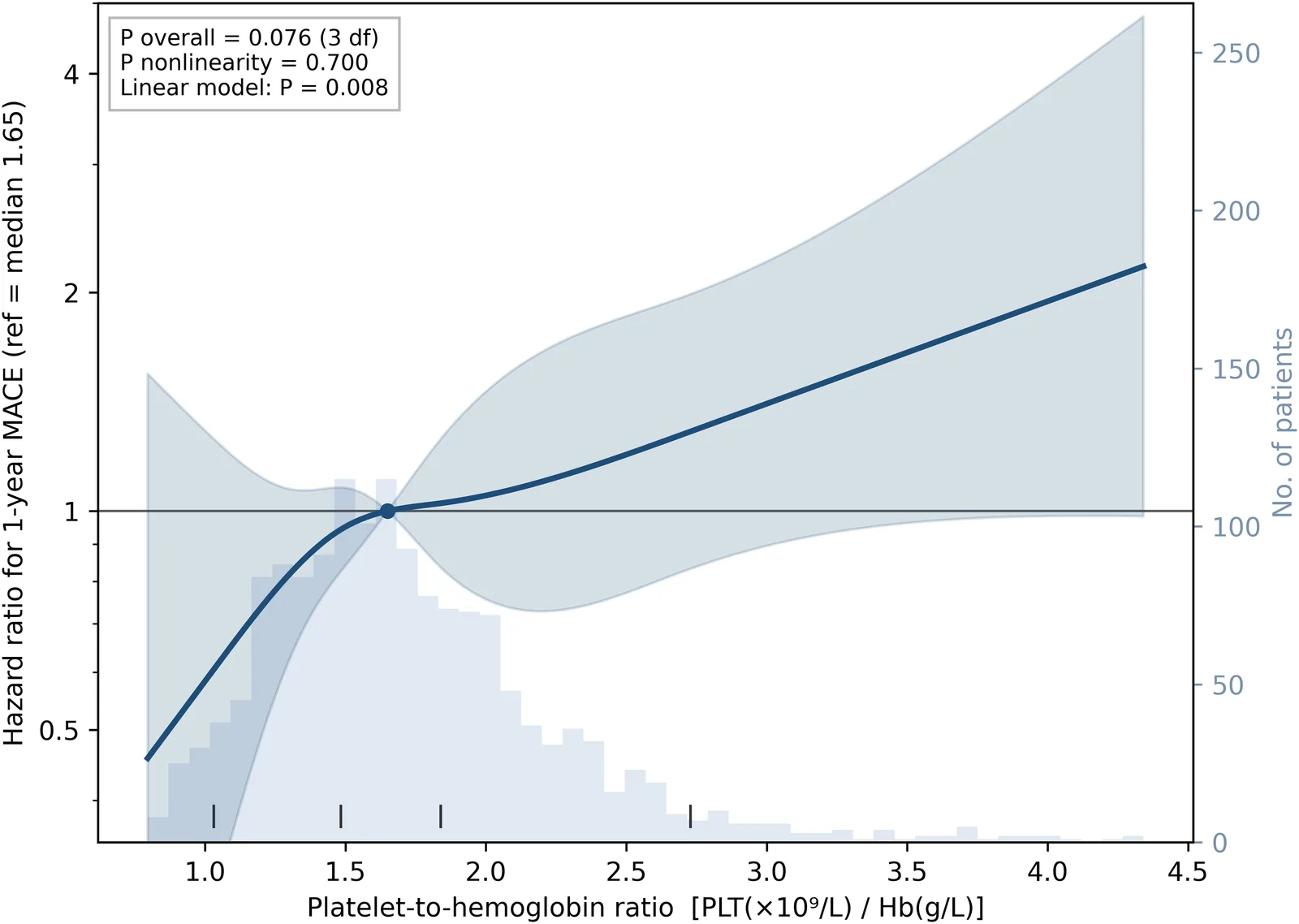
Restricted cubic spline for platelet-to-hemoglobin ratio and 1-year major adverse cardiovascular events. The platelet-to-hemoglobin ratio (PHR) was modeled with a 4-knot restricted cubic spline in a Cox proportional hazards model adjusted for age, sex, hypertension, diabetes mellitus, left main or multivessel disease, myocardial infarction presentation, estimated glomerular filtration rate, and left ventricular ejection fraction. The solid line indicates the hazard ratio relative to the median PHR (1.65, filled circle), with the shaded band representing the 95% confidence interval; the histogram shows the distribution of the PHR. There was no evidence of departure from linearity (P for nonlinearity = 0.700); the overall association of the spline terms was *P* = 0.076 (3 df), whereas the more parsimonious linear model, which yielded a lower Akaike information criterion, showed *P* = 0.008. MACE, major adverse cardiovascular events; PHR, platelet-to-hemoglobin ratio.

### 3.3 Incremental prognostic value and decomposition of the PHR

The model comparisons are summarized in Table 3. Adding the PHR to the clinical model significantly improved model fit (likelihood-ratio *P* = 0.013). Among the untransformed models, the Akaike information criterion was similar for the models containing the PHR (1480.7) and platelet count (1480.4), whereas the log-PHR model yielded the lowest value overall (1480.0). When the components were examined individually, platelet count improved the clinical model (*P* = 0.011) and retained significance after further adjustment for hemoglobin (*P* = 0.013), whereas hemoglobin did not significantly improve the clinical model (*P* = 0.355) or the model already containing platelet count (*P* = 0.473); in the two-component model, the adjusted HRs per 1-standard deviation were 1.24 (95% CI, 1.05–1.46) for platelet count and 0.92 (95% CI, 0.74–1.15) for hemoglobin. On the logarithmic scale, on which the ratio decomposes additively (Table 3, Panel B), the coefficients of log-platelet count (+0.84) and log-hemoglobin (−0.59) were of opposite sign and similar magnitude, and the ratio constraint was not rejected (*χ*^2^ = 0.09, *P* = 0.765); a single log-PHR term retained 98.7% of the likelihood-ratio *χ*^2^ increment of the two-component model, whereas the corresponding proportion on the untransformed scale was 87.8%. The deviation from the constraint (sum of the two log-coefficients) was +0.25 (Wald 95% CI, −1.38 to +1.88; bootstrap 95% CI, −1.35 to +1.99), and the bootstrap distribution of the adequacy index was wide (median, 91.9%; 95% CI, 21.8%–100%), indicating that the data are compatible with the ratio constraint but have limited power to exclude meaningful departures from it. Discrimination improved only marginally with the PHR: the Uno AUC at 365 days was 0.768 for the clinical model and 0.771 with the PHR (*Δ*AUC, +0.003; 95% CI, −0.003 to +0.012; optimism-corrected AUCs, 0.751 and 0.753). Both models were adequately calibrated (optimism-corrected calibration slope, 0.93 and 0.92, respectively), and on decision-curve analysis the net benefit of the model with the PHR was virtually identical to that of the clinical model across threshold probabilities of 5% to 20% (maximum difference, 0.0037, corresponding to 3.7 net true positives per 1,000 patients; Supplementary Figure S1).

**Table 3:** Incremental prognostic value of hemoglobin (Hb), platelet count (PLT), and the platelet-to-hemoglobin ratio (PHR) over the clinical model.

A. Untransformed models					
Models	LR *χ*^2^	AIC	Harrell C	Uno AUC (365 d)	P vs Model A^a^
A: Clinical model	94.05	1484.9	0.757	0.768	
B: Clinical + Hb	94.90	1486.0	0.759	0.770	0.355
C: Clinical + PLT	100.54	1480.4	0.759	0.769	0.011
D: Clinical + Hb + PLT	101.06	1481.8	0.760	0.771	0.030†
E: Clinical + PHR	100.20	1480.7	0.761	0.771	0.013

B. Logarithmic-scale models					
Models	LR χ^2^	AIC	Harrell C	Uno AUC(365 d)	P vs Model A^a^
F: Clinical + log Hb + log PLT	101.01	1481.9	0.761	0.772	0.031^b^
G: Clinical + log PHR	100.92	1480.0	0.761	0.772	0.009

*P* = 0.473; platelet count over clinical + hemoglobin, *P* = 0.013.

In the logarithmic-scale models (Panel B), the ratio constraint (*β* log-platelet = −*β* log-hemoglobin) was not rejected (χ^2^ = 0.09, *P* = 0.765), and the single log-PHR term (Model G) retained 98.7% of the likelihood-ratio χ^2^ increment of the two-component model (Model F) over the clinical model (adequacy index). On the untransformed scale (Panel A), the corresponding proportion was 87.8% (6.16/7.01), reflecting that a ratio index decomposes additively only on the logarithmic scale. The deviation from the constraint (*β* log-platelet + *β* log-hemoglobin) was +0.25 (Wald 95% CI, −1.38 to +1.88; bootstrap 95% CI, −1.35 to +1.99), and the bootstrap 95% CI of the adequacy index was 21.8%–100% (median, 91.9%; 1,000 resamples).

Uno AUC computed by inverse-probability-of-censoring weighting at 365 days. Optimism-corrected AUC (600 bootstrap resamples): Model A 0.751, Model E 0.753; *Δ*AUC (E−A) + 0.003 (95% CI −0.003 to +0.012).

AIC, Akaike information criterion; AUC, area under the receiver operating characteristic curve; CI, confidence interval; df, degrees of freedom; Hb, hemoglobin; LR, likelihood ratio; PHR, platelet-to-hemoglobin ratio; PLT, platelet count.

a Likelihood-ratio test vs the clinical model (1 df).

b 2 df. Additional nested comparisons: hemoglobin over clinical + platelet count.

### 3.4 Joint stratification by anemia and platelet count

When patients were cross-classified by anemia and the median platelet count, the Kaplan–Meier cumulative incidence of MACE at 1 year was 5.6%, 5.0%, and 8.6% in patients with neither feature, a platelet count at or above the median alone, and anemia alone, respectively, and 18.4% in patients with both features (log-rank *P* < 0.001; Figure 3). Compared with patients with neither feature, only the combination of anemia and a platelet count at or above the cohort median was associated with a significantly increased adjusted risk (HR, 1.82; 95% CI, 1.05–3.15; *P* = 0.034; platelet count at or above the cohort median alone: HR, 1.03; 95% CI, 0.63–1.70; anemia alone: HR, 0.63; 95% CI, 0.32–1.22). A multiplicative interaction was observed on the binary scale (*P* = 0.011) but not between continuous hemoglobin and platelet count (*P* = 0.894), suggesting that the apparent interaction may reflect categorization and concentration of risk in the joint high-risk group rather than a continuous multiplicative interaction. Although the unadjusted curves of the two groups with a platelet count below the median crossed (Figure 3), scaled Schoenfeld residuals did not indicate non-proportionality for the group indicators (*P* = 0.25, 0.11, and 0.30 for the higher-platelet-count-only, anemia-only, and combined groups, respectively), and the restricted mean event-free time at 365 days was 27.4 days shorter in the combined group than in patients with neither feature (323.0 vs 350.4 days; 95% CI for the difference, −44.4 to −10.3; *P* = 0.002), supporting the unadjusted group difference without reliance on the proportional hazards assumption (Supplementary Table S2).

**Figure 3:**
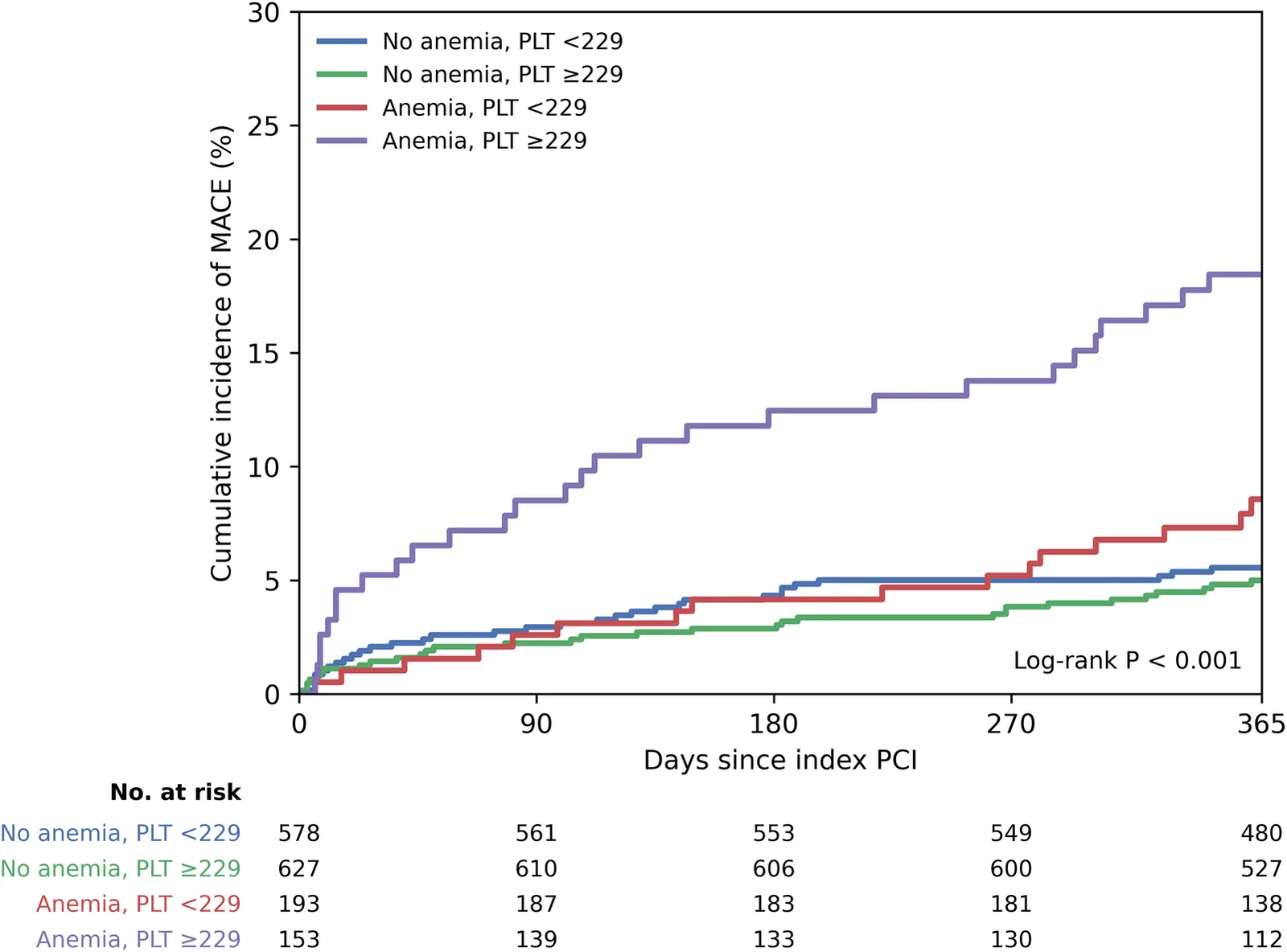
Major adverse cardiovascular events by anemia and platelet-count categories. Patients were classified into four groups according to the presence of anemia (hemoglobin <13 g/dL in men, <12 g/dL in women) and a platelet count below versus at or above the cohort median (229 × 10^3^/*μ*L). Kaplan–Meier curves are displayed through day 365, the primary analytic horizon, with numbers at risk shown at 90-day intervals. The four groups differed significantly (log-rank *P* < 0.001), with the highest event rate in patients with both anemia and a platelet count at or above the median, in whom the Kaplan–Meier estimate at 1 year was 18.4% (versus 5.6% in patients with neither feature) and the adjusted hazard ratio was 1.82 (95% confidence interval, 1.05–3.15; *P* = 0.034) compared with patients with neither feature. MACE, major adverse cardiovascular events; PCI, percutaneous coronary intervention; PLT, platelet count.

### 3.5 Endpoint-specific associations

Exploratory endpoint-specific analyses suggested different component association patterns across outcomes (Figure 4). A higher PHR was associated with all-cause death (adjusted HR per 1-standard deviation, 1.28; 95% CI, 1.03–1.58; *P* = 0.025) and cardiovascular death (HR, 1.28; 95% CI, 1.03–1.58; *P* = 0.025), paralleling the inverse association of hemoglobin with these endpoints. The PHR estimate for any repeat revascularization was directionally positive but did not reach statistical significance (HR, 1.24; 95% CI, 0.97–1.58; *P* = 0.081), whereas platelet count was significantly associated with this outcome (HR, 1.39; 95% CI, 1.07–1.80; *P* = 0.014). No association was observed with stroke, and the association with heart failure hospitalization did not reach significance (HR, 1.32; 95% CI, 0.88–1.99; *P* = 0.175).

**Figure 4:**
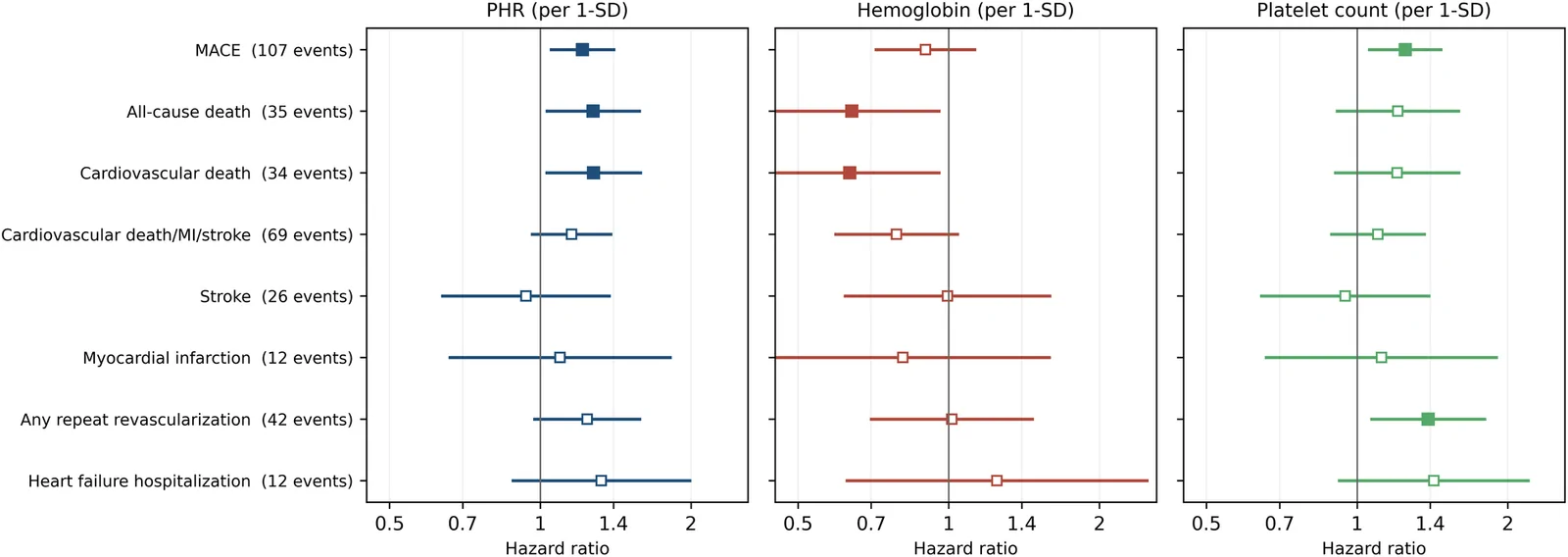
Endpoint-specific associations of platelet-to-hemoglobin ratio and its components. Cause-specific Cox proportional hazards models were fitted for each endpoint with adjustment for age, sex, hypertension, diabetes mellitus, left main or multivessel disease, myocardial infarction presentation, estimated glomerular filtration rate, and left ventricular ejection fraction. Hazard ratios are shown per 1-standard deviation increase in the PHR (0.58; left panel), hemoglobin (1.9 g/dL; middle panel), and platelet count (63 × 10^3^/μL; right panel), with horizontal lines indicating 95% confidence intervals on a logarithmic axis. Filled squares denote *P* < 0.05 and open squares *P* ≥ 0.05. The association of the PHR with mortality endpoints paralleled the inverse association of hemoglobin, whereas its association with repeat revascularization paralleled the positive association of platelet count. MACE, major adverse cardiovascular events; PHR, platelet-to-hemoglobin ratio; SD, standard deviation.

### 3.6 Subgroup analyses

The association between the PHR and MACE was consistent across all prespecified subgroups, including age, sex, clinical presentation, diabetes, discharge P2Y12 inhibitor, renal function, ejection fraction, multivessel disease, and registry, with no significant effect modification (all P for interaction ≥0.35; Figure 5).

**Figure 5:**
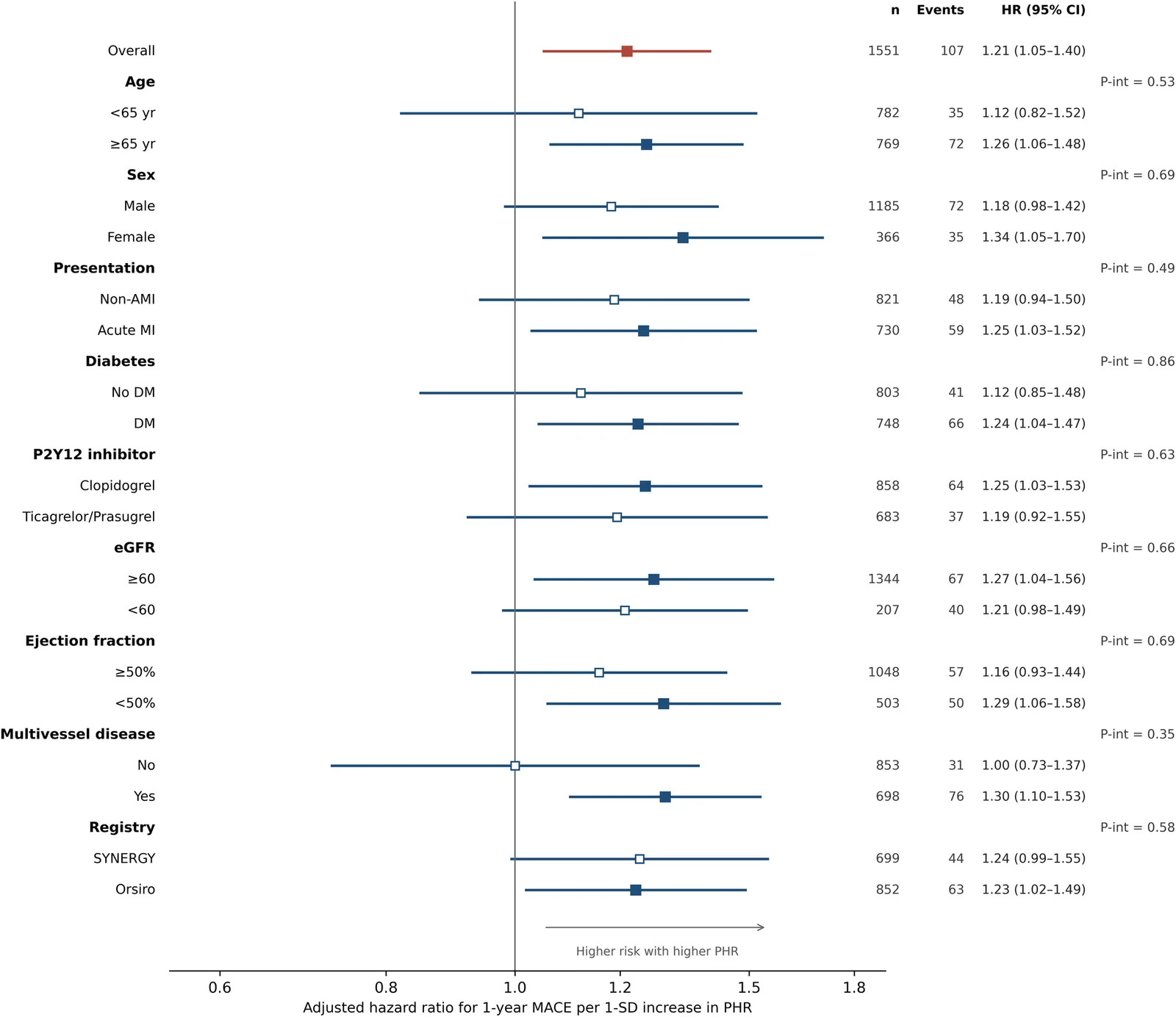
Subgroup analyses of the association between the platelet-to-hemoglobin ratio and 1-year major adverse cardiovascular events. Adjusted hazard ratios (HR) per 1-standard deviation (0.58) increase in the platelet-to-hemoglobin ratio are shown for the overall population (red) and within prespecified subgroups (blue), with horizontal lines indicating 95% confidence intervals (CI) on a logarithmic axis; filled squares denote a lower confidence limit above 1. Cox models were adjusted for age, sex, hypertension, diabetes mellitus, left main or multivessel disease, myocardial infarction presentation, estimated glomerular filtration rate, and left ventricular ejection fraction, with the stratifying variable omitted from the adjustment set within its own strata. P values for interaction were derived from multiplicative interaction terms; no significant effect modification was observed across any subgroup (all P for interaction ≥ 0.35). AMI, acute myocardial infarction; DM, diabetes mellitus; eGFR, estimated glomerular filtration rate; SD, standard deviation.

## 4 Discussion

The main findings of this pooled analysis of two prospective Korean drug-eluting stent registries can be summarized as follows. First, a higher admission PHR was independently associated with 1-year MACE after PCI, and there was no evidence of departure from linearity over the observed range, supporting the use of the PHR as a continuous variable. Second, in the first formal decomposition of this index to our knowledge, the implicit ratio constraint was not rejected and a single log-PHR term retained 98.7% of the two-component likelihood-ratio *χ*^2^ increment in this cohort, supporting the compatibility of the PHR with the ratio constraint and its use as a parsimonious one-degree-of-freedom summary of its components. Third, exploratory analyses suggested endpoint-specific component patterns: the relationship of the PHR with mortality paralleled the inverse association of hemoglobin, whereas its relationship with repeat revascularization paralleled the positive association of platelet count, and absolute risk was concentrated in patients with both anemia and a platelet count at or above the cohort median. Fourth, despite the robust independent association, the incremental gain in discrimination over a conventional clinical model was negligible.

Our findings are broadly consistent with, and extend, the existing PHR literature. Following the initial report from the CORFCHD-PCI study (6), an elevated PHR has been associated with adverse outcomes in coronary artery disease with heart failure (7), STEMI (8), acute coronary syndrome (9), and CABG (10). The present study adds an evaluation in a dedicated all-comer PCI cohort against a broad MACE endpoint with comprehensive multivariable adjustment, in a population and healthcare setting distinct from previous reports. At the same time, two discrepancies deserve comment. Previously proposed cutoff values ranged from 1.69 to 2.2 (7–9); in our cohort, these thresholds fell between approximately the 55th and 82nd percentiles of the PHR distribution, and our upper tertile boundary (1.86) was close to the threshold identified after CABG (1.89) (10). However, given the marked sex difference in the distribution of both components—reflected in the steep sex gradient across tertiles in Table 1—any single cutoff corresponds to substantially different percentiles in men and women, and its transportability across populations is inherently limited. In addition, we did not reproduce the J-shaped relationship reported in some cohorts (7, 9); the association in our data was compatible with a monotonic log-linear gradient. Differences in endpoints (all-cause mortality over several years versus 1-year MACE), in the prevalence of low-PHR states such as thrombocytopenia, and in the use of categorized analyses—which can generate apparent threshold effects from smooth gradients—may account for this discrepancy.

The decomposition analysis carries both methodological and mechanistic implications. Ratio indices embed the strong assumption that their numerator and denominator act with equal and opposite log-linear effects, and when this assumption fails, ratio variables can distort the underlying associations (11). This assumption had not previously been tested for the PHR; in our data, the observed component coefficients were compatible with this assumption, with near-mirror coefficients for log-platelet count and log-hemoglobin and an adequacy index of 98.7%. However, the wide bootstrap confidence interval (21.8%–100%) indicates substantial uncertainty and does not establish that the ratio is equivalent to its components in other populations. Notably, the component contributions were asymmetric after clinical adjustment: platelet count retained independent prognostic information, whereas the contribution of hemoglobin was largely absorbed by age, sex, renal function, and ventricular function—covariates through which the prognostic information of anemia plausibly travels. The endpoint-specific pattern completes this picture and helps reconcile the heterogeneous literature: studies anchored to mortality endpoints (7, 9) predominantly captured the hemoglobin arm of the index, whereas a broad MACE endpoint that includes revascularization, as in the present study, additionally engages the platelet arm. Biologically, the two arms converge on systemic inflammation: interleukin-6 stimulates thrombopoiesis through hepatic thrombopoietin production, driving reactive thrombocytosis (16), while the same inflammatory milieu induces hepcidin-mediated iron restriction and anemia (17). Consistent with this, white blood cell counts and hs-CRP concentrations increased across PHR tertiles in our cohort, and adjustment for these inflammatory markers modestly attenuated the association of the PHR with MACE, consistent with the possibility that part of its prognostic information reflects systemic inflammation. The significant binary interaction between anemia and a platelet count at or above the cohort median, in the absence of any interaction on the continuous scale, should not be interpreted as biological synergy. This discordance may reflect categorization and concentration of risk in the joint high-risk group rather than a continuous multiplicative interaction.

From a clinical standpoint, the appeal of the PHR lies in its simplicity: it is derived at no additional cost from the admission complete blood count, is available for essentially every patient undergoing PCI, and condenses two prognostically relevant axes—oxygen-carrying capacity and thrombopoietic activity—into a single number. The present findings support a prognostic association, but do not establish a role for the PHR as an adjunct to clinical risk stratification or as a threshold-based triage tool. The combination of anemia and a platelet count at or above the cohort median may nevertheless serve as an easily recognizable high-risk phenotype warranting attention to the underlying causes of anemia, the inflammatory burden, and the balance of ischemic and bleeding risk. At the same time, the negligible improvement in discrimination and net benefit indicates that the present data support an independent prognostic association and compatibility with a parsimonious summary of two routine variables, but do not demonstrate a clinically meaningful improvement in risk reclassification; whether the PHR can contribute to decision-making would require evaluation within a formal prediction model and external validation.

Our analysis did not include mean platelet volume (MPV), an index of platelet size that reflects platelet reactivity. In a systematic review with meta-analyses of 41 studies in acute coronary syndrome, admission MPV analyzed as a continuous variable was associated with long-term mortality (HR per unit, 1.33), although not with MACE (18). Platelet count and MPV are physiologically inversely related and capture distinct dimensions of platelet biology—number versus size and reactivity—so the PHR and MPV are unlikely to be redundant, and MPV could act either as a confounder of the association observed here or as complementary prognostic information. Because MPV was not recorded in the registries, we could not examine these relationships, and future studies should evaluate the PHR alongside MPV and other platelet indices within a single model.

This study has several limitations. First, as an observational analysis, residual confounding cannot be excluded despite multivariable adjustment; although the adjustment set was prespecified and estimates were directionally similar across sensitivity analyses, unmeasured factors cannot be ruled out. Second, the PHR was assessed from a single admission sample; in patients with STEMI, acute hemoconcentration may transiently lower the PHR, which may partly explain the lower proportion of STEMI in the highest tertile, and serial changes were not evaluated. Third, although the pooled cohort was derived from prospective multicenter registries, all participants were Korean, the two registries were conducted in sequential periods, and the Orsiro registry preferentially enrolled patients with dysglycemia, which may limit generalizability; estimates were, however, unchanged when the analysis was stratified by registry or center. Fourth, with 107 primary events, the study had limited power to detect modest nonlinearity or meaningful departures from the ratio constraint, as reflected in the wide CI around the constraint deviation and the adequacy index; the compatibility we report should therefore be interpreted as specific to this dataset and does not establish external validity. Fifth, the small event counts for some individual endpoints, particularly MI (12 events) and heart failure hospitalization (12 events), limit precision and increase the risk of overfitting in the adjusted models; these estimates should be interpreted as exploratory. Sixth, bleeding outcomes were not systematically collected, and oral anticoagulant use at discharge was not captured, so that the antithrombotic regimen could be classified only by antiplatelet agents; patients discharged with a P2Y12 inhibitor but without aspirin, almost all in the Orsiro registry, may have received either the P2Y12 inhibitor alone or concomitant oral anticoagulation, which could not be distinguished. Seventh, although the PHR was independently associated with MACE, the negligible gain in discrimination and net benefit means that its clinical utility for risk reclassification has not been established. Eighth, MPV was not recorded in the registries, and its potential confounding or complementary prognostic contribution could not be assessed. Finally, follow-up was limited to 1 year, and the long-term trajectory of the association remains to be defined.

### 4.1 Conclusions

In this pooled analysis of two prospective Korean drug-eluting stent registries, a higher admission platelet-to-hemoglobin ratio was independently associated with 1-year MACE after PCI, with no evidence of departure from linearity. The observed component coefficients were compatible with the ratio constraint, and a single log-PHR term provided a parsimonious statistical summary of their joint prognostic information in this cohort, although the adequacy estimate was imprecise. Exploratory analyses suggested that mortality associations were more closely aligned with hemoglobin and revascularization associations with platelet count; the highest observed risk occurred in patients with both anemia and a platelet count at or above the cohort median. Because the incremental gain in discrimination and net benefit over conventional clinical factors was negligible, the PHR should currently be regarded as an independent, cost-free prognostic marker rather than as a tool for clinical risk reclassification, pending external validation.

## Data Availability

The datasets generated and/or analyzed for this study are not publicly available because of privacy and ethical restrictions. Requests to access the datasets should be directed to the corresponding author and are subject to approval by the Institutional Review Board of Wonkwang University Hospital.
