# Supplementary material for: Admission platelet-to-hemoglobin ratio and 1-year outcomes after percutaneous coronary intervention: a multicenter registry cohort study
Source: Front Cardiovasc Med. 2026 Oct 9;13:1981126. doi: 10.3389/fcvm.2026.1981126 (PMC13690651; doi:10.3389/fcvm.2026.1981126)
Supplement: Supplementary file 1 [file Datasheet1.docx]

**Supplementary appendix**

**Supplement to Byoun JT et al. “Admission platelet-to-hemoglobin ratio and 1-year outcomes after percutaneous coronary intervention: a multicenter registry cohort study”**

**Supplementary table S1. Characteristics of included and excluded patients**

**Supplementary table S2. Assessment of the proportional hazards assumption and analyses not relying on proportional hazards**

**Supplementary table S3. Sensitivity analyses of the association between the platelet-to-hemoglobin ratio and 1-year MACE**

**Supplementary figure S1. Calibration and decision-curve analysis of the clinical model with and without the platelet-to-hemoglobin ratio.**

**Table S1. Characteristics of included and excluded patients**

| **Characteristic** | **Included (n = 1551)** | **Excluded (n = 35)** | **P value** |
| --- | --- | --- | --- |
| Age, years | 64.7 ± 10.8 | 67.0 ± 10.9 | 0.210 |
| Male sex | 1185 (76.4) | 19 (54.3) | 0.005 |
| Diabetes mellitus | 748 (48.2) | 24 (68.6) | 0.027 |
| Acute myocardial infarction | 730 (47.1) | 10 (28.6) | 0.046 |
| STEMI | 378 (24.4) | 3 (8.6) | 0.028 |
| Hemoglobin, g/dL | 13.9 ± 1.9 | 13.5 ± 2.0 | 0.227 |
| Platelet count, ×10³/μL | 236.3 ± 63.0 | 241.9 ± 68.3 | 0.640 |
| PHR | 1.65 [1.37–1.99] | 1.70 [1.43–2.04] | 0.399 |
| Creatinine, mg/dL | 0.90 [0.75–1.07] | 0.89 [0.73–1.03] | 0.562 |
| Orsiro registry | 852 (54.9) | 31 (88.6) | <0.001 |
| MACE within 1 year | 107 (6.9) | 4 (11.4) | 0.302 |

Values are mean ± standard deviation, median [interquartile range], or n (%). P values by Welch's t-test, Mann–Whitney U test, chi-square test, or Fisher's exact test, as appropriate. Patients were excluded solely because of missing model covariates (left ventricular ejection fraction, n = 34, one of whom also lacked a platelet count; hypertension status, n = 1). MACE, major adverse cardiovascular events; PHR, platelet-to-hemoglobin ratio; STEMI, ST-segment elevation myocardial infarction.

**Table S2. Assessment of the proportional hazards assumption and analyses not relying on proportional hazards**

**A. Scaled Schoenfeld residual tests (primary model; rank-transformed time)**

| **Covariate** | **P value** |
| --- | --- |
| PHR (per 1-SD) | 0.707 |
| Age | 0.165 |
| Sex | 0.694 |
| Hypertension | 0.778 |
| Diabetes mellitus | 0.406 |
| Acute MI presentation | 0.029 |
| Left main or multivessel disease | 0.616 |
| eGFR | 0.447 |
| Left ventricular ejection fraction | 0.149 |

**B. Sensitivity analyses for non-proportionality of adjustment covariates**

| **Model** | **HR per 1-SD (95% CI)** | **P value** |
| --- | --- | --- |
| Baseline hazard stratified by MI presentation | 1.21 (1.05–1.40) | 0.008 |
| Baseline hazard stratified by MI presentation and ejection fraction category (<40%, 40–49%, ≥50%) | 1.22 (1.06–1.41) | 0.007 |

**C. Piecewise Cox model with time-varying PHR coefficient**

| **Period** | **Events** | **HR per 1-SD (95% CI)** | **P value** |
| --- | --- | --- | --- |
| Days 0–180 | 70 | 1.20 (1.01–1.43) | 0.041 |
| Days 181–365 | 37 | 1.24 (0.99–1.56) | 0.067 |
| P for difference between periods |  |  | 0.82 |

**D. Restricted mean event-free time (RMST) at 365 days**

| **Group** | **RMST, days (SE)** | **Difference vs reference, days (95% CI)** | **P value** |
| --- | --- | --- | --- |
| PHR tertile 1 (reference) | 352.2 (2.6) |  |  |
| PHR tertile 2 | 350.7 (2.8) | -1.5 (-9.1 to +6.0) | 0.690 |
| PHR tertile 3 | 344.6 (3.2) | -7.6 (-15.8 to +0.6) | 0.068 |
| Neither anemia nor a platelet count at or above the median (reference) | 350.4 (2.7) |  |  |
| Platelet count at or above the median only | 354.1 (2.2) | +3.6 (-3.2 to +10.5) | 0.297 |
| Anemia only | 350.1 (4.4) | -0.3 (-10.3 to +9.7) | 0.956 |
| Anemia and a platelet count at or above the median | 323.0 (8.3) | -27.4 (-44.4 to -10.3) | 0.002 |

Proportional hazards were tested with scaled Schoenfeld residuals. The piecewise model included the same covariates as the primary model with separate PHR coefficients before and after 180 days; the P value for difference compares the two coefficients. RMST denotes the mean event-free time over the 365-day window estimated from the Kaplan–Meier curve, with standard errors from the Greenwood-type formula; differences are unadjusted. Platelet count categories were defined as below versus at or above the cohort median (<229 versus ≥229 ×10³/μL); anemia was defined by World Health Organization criteria.

CI, confidence interval; eGFR, estimated glomerular filtration rate; HR, hazard ratio; MI, myocardial infarction; PHR, platelet-to-hemoglobin ratio; SD, standard deviation; SE, standard error.

**Table S3. Sensitivity analyses of the association between the platelet-to-hemoglobin ratio and 1-year MACE**

| **Analysis** | **n** | **Events** | **Adjusted HR per 1-SD (95% CI)** | **P value** |
| --- | --- | --- | --- | --- |
| Primary model (follow-up censored at 365 days) | 1551 | 107 | 1.21 (1.05–1.40) | 0.008 |
| Baseline hazard stratified by registry | 1551 | 107 | 1.22 (1.06–1.41) | 0.007 |
| Registry added as a covariate | 1551 | 107 | 1.22 (1.06–1.41) | 0.007 |
| Baseline hazard stratified by center (3 centers) | 1551 | 107 | 1.21 (1.05–1.40) | 0.008 |
| Additionally adjusted for smoking, previous PCI, and body mass index | 1540 | 106 | 1.23 (1.06–1.43) | 0.006 |
| Additionally adjusted for aspirin at discharge | 1551 | 107 | 1.22 (1.06–1.41) | 0.007 |
| Additionally adjusted for log white blood cell count | 1463 | 99 | 1.19 (1.03–1.39) | 0.022 |
| hs-CRP subset: model without hs-CRP | 1303 | 97 | 1.20 (1.03–1.39) | 0.016 |
| hs-CRP subset: additionally adjusted for log hs-CRP | 1303 | 97 | 1.16 (1.00–1.35) | 0.054 |
| Sex-standardized PHR (per 1 sex-specific SD) | 1551 | 107 | 1.20 (1.05–1.38) | 0.009 |
| Baseline hazard stratified by ejection fraction category | 1551 | 107 | 1.21 (1.05–1.40) | 0.008 |

Unless stated otherwise, models were adjusted for age, sex, hypertension, diabetes mellitus, acute myocardial infarction presentation, left main or multivessel disease, estimated glomerular filtration rate, and left ventricular ejection fraction, with follow-up administratively censored at 365 days. HRs are per 1-standard deviation (0.58) increase in the PHR, except for the sex-standardized analysis, in which the PHR was converted to a z-score within each sex. The hs-CRP subset comprised patients with an available admission hs-CRP; the model without hs-CRP is shown for the same subset to allow direct comparison.

CI, confidence interval; HR, hazard ratio; hs-CRP, high-sensitivity C-reactive protein; MACE, major adverse cardiovascular events; PCI, percutaneous coronary intervention; PHR, platelet-to-hemoglobin ratio; SD, standard deviation.

Figure S1. Calibration and decision-curve analysis of the clinical model with and without the platelet-to-hemoglobin ratio. (A) Calibration at 365 days. Patients were grouped into deciles of predicted risk of MACE from the clinical model (gray circles) and from the clinical model plus the PHR (blue squares); observed risk was estimated by the Kaplan–Meier method within each decile and is plotted against the mean predicted risk, with vertical bars indicating 95% confidence intervals. The dashed line denotes perfect calibration. Optimism-corrected calibration slopes, obtained by bootstrap resampling (300 replicates), were 0.93 for the clinical model and 0.92 for the model with the PHR. (B) Decision-curve analysis at 365 days. Net benefit is plotted against threshold probability for the strategy of classifying patients as high risk when their predicted risk exceeds the threshold, using the clinical model (gray dash-dot line) or the clinical model plus the PHR (blue solid line), compared with classifying all patients as high risk (gray solid line) or none (horizontal line). Net benefit was calculated as the proportion of true positives minus the proportion of false positives weighted by the odds of the threshold probability, with event probabilities estimated by the Kaplan–Meier method to account for censoring. Across threshold probabilities of 5% to 20%, the maximum difference in net benefit between the two models was 0.0037, corresponding to 3.7 net true positives per 1,000 patients. The clinical model comprised age, sex, hypertension, diabetes mellitus, acute myocardial infarction presentation, left main or multivessel disease, estimated glomerular filtration rate, and left ventricular ejection fraction (n = 1,551; 107 events within 1 year).

MACE, major adverse cardiovascular events; PHR, platelet-to-hemoglobin ratio.
